# Health care needs survey to improve preparedness of community outreach clinics after severe flooding in Mulanje district, Malawi

**DOI:** 10.1371/journal.pgph.0005204

**Published:** 2025-09-30

**Authors:** Joseph Njala, Kelvin Balakasi, Khumbo Phiri, Harrison Chimbaka, Amos Makwaya, Deanna Smith, John Songo, Misheck Mphande, Lloyd Njikho, Jackeline R. Chinkonde, Anteneh Worku, Kathryn Dovel, Sam Phiri, Joep J. van Oosterhout

**Affiliations:** 1 Partners in Hope, Lilongwe, Malawi; 2 University of California Los Angeles, Department of Medicine, David Geffen School of Medicine, Los Angeles, California, United States of America; 3 Ministry of Health, Government of Malawi, Lilongwe, Malawi; 4 UNICEF Malawi Office, Lilongwe, Malawi; 5 USAID, Lilongwe, Malawi; 6 Department of Public Health and Family Medicine, Kamuzu University of Health Sciences, Blantyre, Malawi; PLOS: Public Library of Science, UNITED STATES OF AMERICA

## Abstract

Due to the rising impact of climate change, Malawi has increasingly experienced extreme weather events in the last decade, including flooding that resulted in large-scale displacement of vulnerable populations. In response, the Ministry of Health and partners set up outreach clinics at camps for displaced persons. However, little is known about health care needs of the affected populations. We conducted a cross-sectional survey among individuals aged ≥18 years utilizing health services at mobile outreach clinics at 7 campsites in Mulanje district, set up after flooding caused by cyclone Freddy (2023). We describe demographic characteristics, prevalence of self-reported acute and chronic conditions, depression (PHQ-9 tool), intimate partner violence (IPV) and health service satisfaction. Of 341 participants surveyed, median age was 32 (IQR 23–47) years, and 80.1% were female. Fifty-eight percent were displaced persons, the rest resided close to the camps. Compared to non-displaced residents, displaced individuals significantly more frequently had: no formal education (32.5% vs. 15.3%; p < 0.001); worse self-reported health (41.6% vs. 23.6%; p < 0.001) and respiratory illness (31.0% vs. 20.1%; p = 0.025). They had similar prevalence of chronic heart disease or hypertension (23.9% vs. 20.1%; p = 0.415) and unknown HIV status (1.0% vs. 4.2%; p = 0.163), but lower prevalence of disabilities (5.6% vs. 11.8%; p = 0.039). Similar proportions in both groups screened positive for depression (53.5% vs. 56.3%, p = 0.598; 95.7% had minimal/mild depression symptoms) and IPV (78.2% vs 70.8%, p = 0.225), but sexual violence prevalence was higher in displaced persons (34.5% vs 21.4%, p = 0.225). Low satisfaction with health services was uncommon (11.7%) and significantly associated with male sex and a positive depression score. After severe flooding, mobile outreach clinics were frequented by displaced persons and nearby residents, unable to reach their regular health facility. Given high rates of acute illnesses, chronic conditions, depression and IPV, outreach clinics in these settings require multidisciplinary teams with diverse skills to meet the health needs of the attending population.

## Introduction

Flooding disasters are a severe consequence of climate change and account for close to 50% of all natural hazard-induced disasters [1]. Flooding events arising from tropical cyclones have resulted in large losses of human life globally and have led to socio-economic instabilities [2]. In the last two decades, flooding has affected 2.3 billion people’s health outcomes worldwide [3]. In sub-Saharan Africa (SSA), a surge in water- and vector-borne diseases has been observed among displaced populations in the aftermath of flooding events, including malaria and diarrheal diseases such as cholera [2,3]. However, the understanding of short- and long-term effects of flooding, in particular in relation to mental health and chronic diseases, including non-communicable conditions, is still limited [4].

Over the past decade, Malawi has been experiencing perennial flooding disasters, the worst of these occurred in 2023 due to cyclone Freddy [5,6]. The floods in 2023 affected 13 out of the country’s 29 districts and resulted in an estimated 127,000 displaced households, more than 500 deaths, more than 500 missing persons and more than 1,700 injuries, country-wide [6,7]. The southern Mulanje district was one of the worst affected [8].

Displaced populations in Malawi faced challenges in accessing all forms of essential health services, as flooding rendered access routes to health facilities impassable, damaged health facility buildings and equipment and unsettled supply chains. The displacement of households resulted in disruption of social structures, disease outbreaks and treatment interruptions for individuals with chronic health conditions, including HIV, putting extra pressure on the already weak health system [7].

The provision of optimal medical outreach services to relief camps is challenged by the sparse current knowledge about health needs of displaced people in such settings in Malawi, which has limited the preparedness of outreach teams. To better tailor the services to the target population, including knowing which mobile tools and equipment to include, we conducted a survey among individuals seeking care at outreach clinics at several relief camps in Mulanje district, Malawi, collecting information on demographic characteristics, prevalent medical conditions (including chronic diseases) and client satisfaction with the outreach clinics services rendered at the time.

## Materials and methods

### Setting

The 2022 World Bank Poverty Assessment report indicates that half of Malawi’s population is living below the poverty line [9]. Over the last decade, successive occurrences of flooding, droughts, and other natural hazard-induced disasters related to climate change have resulted in compounding poverty drifts [10]. The flooding in Mulanje district caused by cyclone Freddy resulted in 143 deaths, 223 missing persons, around 17,000 displaced households and more than 114,000 displaced individuals, who were temporarily housed in 130 camps. The implementation of this survey took place in seven of these camps, established by the Malawi government in schools and churches that had withstood the floods in the catchment areas of Muloza and Kambenje Health Centres, in Mulanje district. The seven camps housed approximately 1,000 individuals. Soon after the landfall of cyclone Freddy and the resulting flooding, ad hoc weekly, mobile outreach clinics were organized for the camps, coordinated by the district health services of the Ministry of Health (MOH) and by Partners in Hope (PIH), a Malawian, non-governmental, faith-based medical organization that supports HIV services at 22 health facilities in Mulanje district. PIH has experience in offering HIV and TB care with integrated services for non-communicable diseases and other general health care. Before cyclone Freddy, PIH also supported flooding relief efforts by the Malawi government. In the aftermath of cyclone Freddy, PIH provided screening, curative, preventative and referral outreach services. Residents of villages surrounding the camps, who faced difficulties to access regular health facilities due to road infrastructure damage from flooding, also benefited from these outreach clinics. Services offered during the outreach clinics included antenatal care (ANC), family planning, under-5 clinic services, HIV testing services, anti-retroviral therapy (ART) refills, HIV pre-exposure prophylaxis (PrEP), condom distribution, and general primary care. Services were provided by nurses, clinical officers, counselors and HIV testing staff, using basic, portable equipment.

### Participant selection, inclusion criteria, sample size

Individuals presenting to the outreach clinics at camp sites were referred to trained research assistants (RAs) by clinic staff. An opportunity sample was taken by offering enrolment into the survey, by RAs, to every third person attending the outreach clinics to access out-patient services. Only adults, aged 18 years or older, were included. RAs determined eligibility and introduced the survey to eligible individuals. After obtaining written informed consent, participants underwent the survey interview, which was conducted individually with the clinic attendees in a secluded area or room where participants would not be observed or overheard.

### Survey design, data collection, and outcomes

#### Data collection.

We conducted a descriptive, cross-sectional survey. Data was collected from 1 to 30 November 2023 using a structured questionnaire that we developed based on studies done in similar settings [11,12]. The survey covered several domains, including socio-demographic information and participants’ health status. The health status assessment included self-reported physical well-being and mental health, with a specific focus on depression screening using the locally validated PHQ-9 tool [13]. We collected data on self-reported disability, defined as having a physical, hearing, visual or other impairment. The survey also evaluated the prevalence of intimate partner violence (IPV), using the World Health Organization’s validated gender-based violence (GBV) screening tool [14]. Additionally, the questionnaire collected self-reported data on HIV status, TB and respiratory symptoms, diarrhea, hypertension and heart disease, and diabetes mellitus, using screening questions from Malawi MOH program tools [15]. To support participant’s self-reported information, we extracted information from participant’s health passports, if available [16]. Participant screening, verbal consent, and the survey were conducted one-on-one in local language, in a private area. The survey was administered with Android tablets using the SurveyCTO data collection application.

#### Covariates and outcomes.

We categorized study participants into two groups: non-displaced residents and displaced persons. Residents were non-displaced inhabitants of villages in the camp catchment area. Displaced persons were relocated to the camp from their flooded areas of residence. We considered that health care needs may be different for the two groups, mainly because they endured different hardships and were residing in different conditions, which may cause variable impact on health and illness.

We established that a participant had “acute respiratory symptoms” if they reported any of the following: cold, runny nose, fever, cough, sore throat or pneumonia. We dichotomized those with a positive PHQ-2 score, into two categories: minimal/mild depression (PHQ-9 score of 1–9) and moderate/severe depression (PHQ-9 score of ≥10).

Marital status of participants was dichotomized as single (including divorced, separated and widowed) vs. married/steady relationship. We defined steady relationship as relationships where the participant and their partner were cohabiting. Among married/cohabiting participants, we defined IPV as positive if the participant reported having experienced any emotional-, physical- or sexual violence and/or controlling behavior from their partners at least once in the past 3 months. We did not determine IPV outside of steady relationships. We considered that the prevalence of IPV may be high in camps for displaced persons, where stress from experiencing hardships and overcrowding may increase interpersonal tensions and where security is limited.

Lastly, we assessed client satisfaction with outreach clinic services using a modified version of the validated Patient Satisfaction Questionnaire short form (PSQ-18) [17]. To improve outreach clinic service preparedness, we used this validated tool to gain insight into several aspects of health services that may need attention for a better patient experience. We only used 10 questions from PSQ-18 that were deemed relevant to the local setting, informed by similar work done in Malawi [18], including about waiting time, attention of healthcare workers for patients’ concerns, opportunity to ask questions, healthcare workers’ politeness, explanations given, privacy, availability of medications, convenience of opening times, cleanliness-hygiene and unmet health concerns. We excluded a question about costs, as services were provided free of charge and transport costs were unlikely. Clients were asked to rate which aspects of care received that day were problematic for them using a three-point scale: no problem, minor problem and major problem. Clients also had an option to provide no answer. At analysis, scores were inversely recoded such that no problem got the highest score of 3 while major problem got the lowest score of 1. Providing no answer was assigned a score of 2, i.e., neutral. A total score was created by summing up the scores for each of the 10 components and then a dichotomized score was created, with scores 10–20 coding low satisfaction and 21–30 high satisfaction.

### Data analysis

We used descriptive statistics to understand participant demographic characteristics, general health findings, prevalence of specific health conditions and client satisfaction. Differences between displaced and resident participants were assessed using chi-square tests for categorical variables and Kruskal-Wallis for equality of medians of continuous variables. In regression analysis, we assessed factors associated with the dichotomized client satisfaction score being low. We estimated a negative binomial risk regression model because low service satisfaction was common (>10%). All variables associated (p < 0.1) with low satisfaction from crude regressions, plus age, were included in multivariable regression analysis to assess the effects of the variables adjusting for demographic characteristics. IPV was not considered for this analysis as only cohabiting couples underwent IPV screening. Standard errors in crude and adjusted logistic regressions were adjusted to account for clustering by outreach clinic service point. Statistical significance was assessed at p < 0.05. All statistical analyses were conducted in STATA/MP 18.0 (StataCorp LLC, Texas, USA).

### Ethical considerations

This study was conducted with approval from the National Health Sciences Research Committee in Malawi (#23/09/4180). All participants provided written informed consent prior to their involvement in the study. To compensate opportunity costs, participants received an equivalent of USD 5.

## Results

### Characteristics of outreach clinic attendants

We enrolled 341 clients who presented at the outreach clinics. 57.8% (197/341) were displaced persons and 42.2% (143/341) resided close to the camps (residents). The median age was 32 years (IQR 23–47) and 80.1% were female. Compared to residents, displaced people more often had a lower level of education, were more frequently in a female-headed household and had different employment status (more frequently farming). There were no significant differences between the groups in sex, median age, marital status and in the median number of living children (Table 1).

**Table 1:** Sociodemographic characteristics of outreach clinic attendants.

Variable	Total	Resident^1^	Displaced	p-value
	n = 341	n = 144	n = 197	
Sex, % (n)				
Female	80.1 (273)	77.8 (112)	81.7 (161)	
Male	19.9 (68)	22.2 (32)	18.3 (36)	0.367
*Among females (n = 273)*				
Pregnant, % (n)				
No	94.5 (258)	93.8 (105)	95.0 (153)	
Yes	5.5 (15)	6.2 (7)	5.0 (8)	0.648
Household type, % (n)				
Female-headed	27.9 (95)	22.2 (32)	32.0 (63)	
Male-headed	70.4 (240)	77.1 (111)	65.5 (129)	0.049
Missing	1.8 (6)	0.7 (1)	2.5 (5)	
Age, median (IQR)	32.0 (23-47)	31.5 (23-50)	32.0 (23-46)	0.822^
Age group, % (n)				
18-24 years	30.8 (105)	30.6 (44)	31.0 (61)	
25-40 years	36.7 (125)	37.5 (54)	36.0 (71)	
41 years or older	32.6 (111)	31.9 (46)	33.0 (65)	0.960
Highest level of education completed, % (n)				
None	25.2 (86)	15.3 (22)	32.4 (64)	
Primary	60.4 (206)	73.6 (106)	50.8 (100)	
Secondary or higher	14.4 (49)	11.1 (16)	16.8 (33)	<0.001
Marital status, % (n)				
Single/not married	37.2 (127)	37.5 (54)	37.1 (73)	
Married/steady relationship	62.8 (214)	62.5 (90)	62.9 (124)	0.933
Number of living children, median (IQR)	3.0 (1–4)	3.0 (1–4)	3.0 (1–5)	0.074^
Primary occupation, % (n)				
Farming	74.2 (253)	66.0 (95)	80.2 (158)	
Other	25.8 (88)	34.0 (49)	19.8 (39)	0.003

^1^ *Resided within the catchment area of the camp, not displaced due to flooding; ^Kruskal-Wallis p-value of equality of medians.*

*IQR, interquartile range.*

### Self-reported health status

Overall, one-third of clients of outreach clinics considered their general health status as poor or very poor, which was significantly higher among displaced people than among residents. Displaced persons also reported a significantly higher number of days that they had been sick in the last month and significantly more acute respiratory symptoms. Residents had a significantly higher prevalence of disabilities. Overall, 127 (37.2%) had a composite of 3 chronic conditions (heart disease/hypertension, diabetes, HIV), 77 (39.1%) displaced persons and 50 (34.7%) residents (p = 0.410).

There were no significant differences in self-report of feeling sick on the survey day, having diarrhea, having TB symptoms, having chronic heart disease or hypertension, and in HIV status between displaced clients and residents. All people living with HIV (PLHIV) in both groups were engaged in HIV care and registered on ART (Table 2).

**Table 2:** Health status of outreach clinic attendants.

Variable	Total	Resident^1^	Displaced	p-value
	n = 341	n = 144	n = 197	
Self-rated general health, % (n)				
Poor/very poor	34.0 (116)	23.6 (34)	41.6 (82)	
Good/very good	66.0 (225)	76.4 (110)	58.4 (115)	<0.001
Days too sick to work last month, % (n)				
None	43.7 (149)	50.7 (73)	38.6 (76)	
1-3 days	27.3 (93)	20.1 (29)	32.5 (64)	
4 or more days	29.0 (99)	29.2 (42)	28.9 (57)	0.024
Feeling ill on the survey day, % (n)				
No	5.3 (18)	6.9 (10)	4.1 (8)	
Yes	94.7 (323)	93.1 (134)	95.9 (189)	0.240
Acute respiratory symptoms, % (n)				
No	73.6 (251)	79.9 (115)	69.0 (136)	
Yes	26.4 (90)	20.1 (29)	31.0 (61)	0.025
Diarrhea, % (n)				
No	89.7 (306)	88.9 (128)	90.4 (178)	
Yes	10.3 (35)	11.1 (16)	9.6 (19)	0.659
Presumptive TB symptoms, % (n)				
No	92.6 (299)	95.5 (128)	90.5 (171)	
Yes	7.4 (24)	4.5 (6)	9.5 (18)	0.088
HIV status, % (n)				
HIV positive	18.8 (64)	18.8 (27)	18.8 (37)	
HIV negative	78.9 (269)	77.1 (111)	80.2 (158)	
Unknown status	2.3 (8)	4.2 (6)	1.0 (2)	0.163
Chronic heart disease or hypertension, % (n)				
No	77.7 (265)	79.9 (115)	76.1 (150)	
Yes	22.3 (76)	20.1 (29)	23.9 (47)	0.415
Diabetes, % (n)				
No	99.4 (339)	99.3 (143)	99.5 (196)	
Yes	0.6 (2)	0.7 (1)	0.5 (1)	0.823
Skin condition, % (n)				
No	94.1 (321)	95.8 (138)	92.9 (183)	
Yes	5.9 (20)	4.2 (6)	7.1 (14)	0.254
Disability^, % (n)				
No	91.8 (313)	88.2 (127)	94.4 (186)	
Yes	8.2 (28)	11.8 (17)	5.6 (11)	0.039

^1^*Resided within the catchment area of the camp; had not been displaced due to flooding; ^Defined as having a physical, hearing, visual or other impairments*.

### Depression, intimate partner violence and service satisfaction

Similar proportions in both groups screened positive for depression. Overall, less than 5% had moderate depression, none had severe depression. More than three-quarters of the participants had experienced IPV in the last 3 months. Overall prevalence of physical IPV was 31.3% and of sexual IPV 28.9%. Sexual IPV was significantly higher among displaced people compared to residents (34.5% vs 21.4%; p = 0.039). Neither the overall prevalence of IPV nor any of the three other forms of IPV were significantly different between displaced people and residents. The overall prevalence of IPV (76.2% vs. 70.5%; p = 0.433) was similar between women and men, but sexual abuse (32.9% vs. 13.6%; p = 0.012) was significantly more common among women. A small minority of participants (11.7%) had a low client satisfaction score, with no significant difference between displaced persons and residents (Table 3).

**Table 3:** Depression, IPV and service satisfaction.

Variable	Total	Resident^1^	Displaced	p-value
	n = 341	n = 144	n = 197	
Depression screening (PHQ2 score), % (n)				
Negative	44.9 (153)	46.5 (67)	43.7 (86)	
Positive	55.1 (188)	53.5 (77)	56.3 (111)	0.598
*Among PHQ2 screened positives (n = 188), % (n)*				
Minimal/mild	95.3 (325)	94.4 (136)	95.9 (189)	
Moderate/severe*	4.7 (16)	5.6 (8)	4.1 (8)	0.519
IPV screening (n = 208), % (n)				
Emotional abuse	38.5 (80)	36.0 (32)	40.3 (48)	0.521
Physical abuse	31.3 (65)	30.3 (27)	31.9 (38)	0.806
Sexual abuse	28.9 (60)	21.4 (19)	34.5 (41)	0.039
Controlling behaviour	54.3 (113)	55.1 (49)	53.8 (64)	0.855
Overall	75.0 (156)	70.8 (63)	78.2 (93)	0.225
Client satisfaction score, % (n)				
Low satisfaction	11.7 (40)	14.6 (21)	9.6 (19)	
High satisfaction	88.3 (301)	85.4 (123)	90.4 (178)	0.162

^1^Resided within the catchment area of camps, not displaced due to the floods. *None had a score of severe depression.

**Fig 1:**
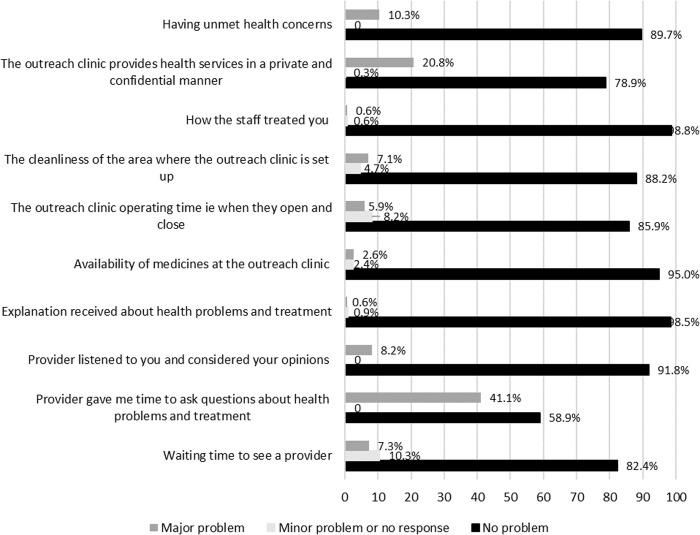
Satisfaction with health services among 341 clients of mobile clinics.

Fig 1 provides an overview of the ten questions that contributed to the client satisfaction score. While overall satisfaction with the health services appeared high, most problems were expressed with privacy/confidentiality and with the opportunity to ask questions about health problems and treatment.

### Factors associated with a low client satisfaction score

We assessed the association between a low score of satisfaction with health services provided at outreach clinics and sociodemographic factors, health status and depression. In crude risk regression analysis, male sex, older age, having had primary education (vs. no formal education), poor self-reported health status and a positive depression score were associated with low satisfaction (p < 0.1) (Table 4).

In multivariate risk regression, sex and depression score remained independently associated with low satisfaction with the health services at outreach clinics. Females were less than half as likely to have low satisfaction (aOR 0.42, 95%-CI 0.22-0.80; p-value 0.009). Individuals who had a minimal, mild or moderate result in the depression score were more than 3 times as likely to have a low satisfaction score (aOR 3.14, 95%-CI 1.55-6.36; p-value 0.001) than those with a negative PHQ-2 result

**Table 4:** Factors associated with low satisfaction with health services among clients of outreach clinics.

Variable	Total	RR	95% CI	p-value	aRR	95% CI	p-value
	% (n = 341)						
Displaced							
No	42.2 (144)	Ref					
Yes	57.8 (197)	0.66	0.31-1.41	0.286			
Sex							
Male	19.9 (68)	Ref			Ref		
Female	80.1 (273)	0.37	0.21-0.65	0.001	0.42	0.22-0.80	0.009
Age group							
18-24 years	30.7 (105)	Ref			Ref		
25-40 years	36.7 (125)	1.26	0.75-2.11	0.379	0.88	0.41-1.89	0.742
41 years or older	32.6 (111)	2.36	1.13-4.95	0.022	1.74	0.73-4.17	0.212
Highest level of education completed							
None	25.2 (86)	Ref			Ref		
Primary	60.4 (206)	0.50	0.33-0.76	0.001	0.72	0.39-1.33	0.291
Secondary or higher	14.4 (49)	0.82	0.31-2.16	0.686	1.12	0.29-4.37	0.869
Marital status, % (n)							
Single	37.2 (127)	Ref					
Married/steady relationship	62.8 (214)	0.99	0.38-2.56	0.982			
Number of children							
None	8.2 (28)	Ref					
1-3 children	52.8 (180)	0.62	0.28-1.36	0.235			
4 or more children	39.0 (133)	0.63	0.34-1.19	0.153			
Primary occupation							
Other	25.8 (88)	Ref					
Farming	74.2 (253)	0.81	0.38-1.76	0.596			
Self-rated health							
Good/very good	66.0 (225)	Ref			Ref		
Poor/very poor	34.0 (116)	2.14	1.66-2.77	0.001	1.39	0.92-2.08	0.114
HIV status							
HIV negative/unknown	81.2 (277)	Ref					
HIV positive	18.8 (64)	1.44	0.67-3.09	0.346			
Acute respiratory symptoms							
No	73.6 (251)	Ref					
Yes	26.4 (90)	0.70	0.30-1.62	0.401			
Chronic heart disease or hypertension							
No	77.7 (265)	Ref					
Yes	22.3 (76)	1.16	0.77-1.75	0.471			
Among those screened PHQ-9 score							
None	44.9 (153)	Ref			Ref		
Minimal/mild + Moderate	55.1 (188)	3.26	1.61-6.59	0.001	3.14	1.55-6.36	0.001

## Discussion

In this cross-sectional health survey at outreach clinics for relief camps that were set up in the aftermath of cyclone Freddy, we found that a large part (80.1%) of the 341 adult clients was female and around 4 in 10 were non-displaced residents from areas around the camps. Compared to these residents, displaced persons were more often part of a female-headed household, had lower education levels and were more frequently farmers. Displaced persons rated their general health more poorly, reported being ill in the last month more frequently and reported respiratory illness more commonly. The overall prevalence rates of IPV (75.0%) and depressive symptoms (55.1%) were high, but not significantly different between the 2 groups and depression scores were mostly minimal or mild. A low score of satisfaction with the outreach clinic services was uncommon (11.7%), and was significantly associated with male sex and a positive PHQ-2 depression score.

The composition of the adults utilizing outreach health care services at camps for displaced persons and their clinical presentations provide direction for outreach service requirements. The vast majority were women, of whom 5.5% were pregnant, which indicates the need for availability of family planning services and basic gynecological, antenatal and obstetric care, especially since adverse pregnancy outcomes are common after flooding events [19]. Some 40% of patients utilizing outreach health services were actually not residing in the camps where the outreach clinics took place. Since the service was conveniently offered close to the homes of residents, free of charge and open to all, this was not a surprise and is also of justified benefit for residents, many of whom could not reach their regular health facility due to the damage to roads infrastructure by flooding. Apart from a poorer reported general health status and higher presence of acute respiratory symptoms among displaced persons, the health status seemed similar between the 2 groups. The fact that residents more often reported disabilities may be due to the reduced capacity of persons from flooded areas with disabilities to reach relief camps.

A systematic review that included 10 studies from sub-Saharan Africa and addressed health conditions of victims of flooding disasters found that such individuals had an increased risk of several vector- and waterborne infectious diseases, including cholera, scabies, taeniasis, east-African trypanosomiasis, malaria and alpha- and flavivirus infections [19]. Another report indicated that the risk of leptospirosis is increased after flooding events [20]. Common immediate health risks during flooding concern (near-) drowning, injuries and sometimes intoxications from accidentally released chemicals [21]. We did not report such conditions, likely due to the fact that our survey took place many weeks after the initial landfall of cyclone Freddy. The timing of our survey has impact on the observed conditions, in particular the acute diseases, such as diarrheal diseases and respiratory infections, which may be higher in the immediate aftermath of a flooding disaster. Our data may therefore more reflect the health needs of displaced persons who form a secondary surge of casualties who have a higher burden of chronic conditions that can overwhelm a health system [22].

Around 10% of the adult patients at the outreach clinics had a diarrheal disease, which may be similar to the prevalence at population level in Malawi, estimated at 18% [23], but the latter included children who have a higher burden of diarrhea. As Malawi is a cholera endemic country [24] and given that cholera outbreaks often occur during flooding in overcrowded settings with poor hygiene [19], cholera diagnostics need to be available and there needs to be preparedness for isolating suspected and confirmed cases at dedicated cholera management areas [25]. 7.4% overall and 9.5% among displaced persons had a positive WHO TB-symptom screen, comparable to the estimated prevalence of TB symptoms of 8.6% in the national population [26]. This argues for making diagnostics for follow-up TB testing available, including mobile X-ray equipment [27] and portable molecular TB testing [28].

Little attention has been given to the impact of flooding disasters on chronic diseases [19], yet the prevalence of chronic conditions in our survey was high. Close to one-in-five patients were living with HIV, higher than the adult national prevalence, which was 6.2% in 2024 [29], and all were on ART, thus prone to detrimental interruptions in treatment, that can lead to deterioration of the individual health status, development of HIV drug resistance [30] and transmission of HIV [31]. Regular ART services at outreach clinics, including multi-month dispensing and viral load sample transport or portable molecular viral load equipment are therefore paramount to prevent disruption of HIV care and treatment [31]. More than one-in-five clinic attendants had hypertension or chronic heart disease, which is in line with the prevalence of these conditions in Malawi [32,33]. Outreach clinics therefore need to be equipped with adequate blood pressure measurement equipment and be able to provide medication for cardiovascular diseases, to avoid interruptions of treatment that could evoke complications, including unstable angina pectoris, myocardial infarction, stoke and renal insufficiency [34].

Just over half the patients screened positive for depression, which may have been expected given the dire circumstances that many had experienced during and after the flooding, including deaths of friends and relatives, and loss of possessions and livelihoods. Mental health problems were common among adults in other settings in the aftermath of natural disasters, with depression prevalence among adults ranging from 6% to 54% in a systematic review from 2014 [35]. Other common mental health problems after flooding are anxiety and post-traumatic stress disorder [36], which we did not screen for. Because nearly all individuals with a positive depression screen had minimal or mild symptoms, counseling by a trained lay cadre staff in the outreach clinic team may be an adequate and sufficient intervention for most [37,38]. In addition, preventative mental health measures that are integrated in general disaster preparedness may benefit mental health in areas at high risk of flooding [36].

We found that IPV was highly common among patients who are married or cohabiting. Observations from Uganda, Zimbabwe, Mozambique and Kenya have suggested that extreme weather events are associated with IPV among women [39,40], although we did not find documentation of IPV from Africa specifically during flooding. Of the 4 domains of IPV, sexual violence was the only that was significantly more common among women and among displaced persons. These findings indicate that post-IPV interventions need to be available at outreach clinics and that IPV prevention measures are required in post-flooding relief settings in Malawi. Research on the prevention of IPV in low- and middle-income countries has focused on longer-term interventions [41,42] and as far as we know not on the specific circumstances described here during flooding, indicating an evidence gap.

Only 12% of the patients expressed low satisfaction with the services provided, with clients having most problems with a lack of privacy/confidentiality and with limited opportunities to ask questions about health conditions and treatments. We could not find literature references on service satisfaction surveyed at outreach clinics in similar circumstances. A study from Pakistan found that satisfaction with the general disaster relief response (i.e., not specific for health care) after a flooding event among affected residents was low and longer-term satisfaction was associated with the availability of medicines [43]. Although our survey did not enquire about this, the lack of interventions for depressive symptoms and for IPV may have played a role in low service satisfaction, given the high prevalence of these problems. This was underlined by the association of depression with lower satisfaction with the outreach clinic services.

Our study had a number of limitations. We did not include children and cannot provide recommendations for this important group of affected individuals with specific needs. We relied on self-reported symptoms and conditions and there may have been inaccuracies and biases resulting from this. Although RAs assured participants about the privacy of the survey and that participation would not have any consequences for current and future care at the outreach clinic, bias could have occurred due to existing power dynamics between patients and survey staff/ health care providers. Furthermore, selection bias is likely as the survey was taken at the point of care, which would have excluded individuals who could not access the outreach clinic. We did not collect detailed information about how the flooding had affected individual lives, for instance through the loss of a relative, while this may have repercussions for the mental health status. The geographical scope of our survey was limited and although we believe that circumstances were similar in other affected areas in Malawi, care must be taken with extrapolation of our findings to other settings. Lastly, we did not enquire IPV experience from individuals who were not married or cohabiting, which gave an incomplete insight into IPV prevalence and precluded determining whether experience of IPV was associated with outreach clinic service satisfaction.

## Conclusion

After severe flooding, mobile outreach clinics were frequented by displaced persons in camps and also by nearby residents who were unable to reach their regular health facility. Given high rates of acute illnesses, chronic conditions (including HIV and non-communicable diseases), depression and IPV, mobile clinics in these settings require multidisciplinary teams with diverse skills and mobile equipment to provide satisfactory health services to the attending patient population. Paying attention to privacy and allowing clients to ask questions about their health may further improve satisfaction with the outreach clinic services. Larger studies in more varied settings and timed sooner after the onset of a flooding disaster are needed for comprehensive insights into displaced flooding victims’ health needs, especially those related to chronic conditions.

## Supporting information

S1 Table Anonymized data. (XLSX)
